# TDP-43 as a possible biomarker for frontotemporal lobar degeneration: a systematic review of existing antibodies

**DOI:** 10.1186/s40478-015-0195-1

**Published:** 2015-04-01

**Authors:** Joery Goossens, Eugeen Vanmechelen, John Q Trojanowski, Virginia MY Lee, Christine Van Broeckhoven, Julie van der Zee, Sebastiaan Engelborghs

**Affiliations:** Reference Center for Biological Markers of Dementia, Laboratory of Neurochemistry and Behavior, Institute Born-Bunge, University of Antwerp, Universiteitsplein 1, 2610 Wilrijk, Belgium; ADx NeuroSciences, Technologiepark 4, 9052 Ghent, Belgium; Center for Neurodegenerative Disease Research & Institute on Aging, Department of Pathology and Laboratory Medicine, Perelman School of Medicine, University of Pennsylvania, Philadelphia, PA 19104-4283 USA; Neurodegenerative Brain Diseases Group, Department of Molecular Genetics, VIB, Universiteitsplein 1, 2610 Wilrijk, Belgium; Laboratory of Neurogenetics, Institute Born-Bunge, University of Antwerp, Universiteitsplein 1, 2610 Wilrijk, Belgium

**Keywords:** TDP-43, Antibodies, Immunoassay, Biomarkers, Frontotemporal lobar degeneration (FTLD)

## Abstract

Frontotemporal lobar degeneration (FTLD) is one of the leading causes of dementia after Alzheimer’s disease. A high-ranking candidate to become a diagnostic marker for a major pathological subtype of FTLD is the transactive response DNA binding protein of 43 kDa (TDP-43). The main objective is to elucidate which antibodies are specific for pathological TDP-43, with special interest in its modified isoforms. Indeed, TDP-43 has been shown to be hyperphosphorylated and truncated in disease. A secondary objective is to review existing immunoassays that quantify TDP-43 in biofluids. A systematic review of literature was performed by searching PubMed and Web of Science using predefined keywords. Of considered research papers the methods section was reviewed to select publications that enabled us to answer our learning objective. After quality assessment, antibody characteristics and related outcomes were extracted. We identified a series of well-characterized antibodies based on a scoring system that assessed the ability of each antibody to detect TDP-43 pathology. A selection of 29 unique antibodies was made comprising 10 high-ranking antibodies which were reported multiple times to detect TDP-43 pathology in both immunostaining and immunoblotting experiments and 19 additional antibodies which detected TDP-43 pathology but were only scored once. This systematic review provides an overview of antibodies that are reported to detect pathological TDP-43. These antibodies can be used in future studies of TDP-43 proteinopathies. Additionally, selected antibodies hold the potential to be used in the development of novel immunoassays for the quantification of TDP-43 in biofluids, as a possible biomarker for FTLD-TDP.

**Electronic supplementary material:**

The online version of this article (doi:10.1186/s40478-015-0195-1) contains supplementary material, which is available to authorized users.

## Introduction

Frontotemporal lobar degeneration (FTLD) is the primary cause of early onset dementia after Alzheimer’s disease (AD) [1]. Worldwide prevalence of FTLD is underestimated due to difficult diagnosis complicated by clinical, neuropathological and genetic heterogeneity [2-5]. FTLD is clinically subdivided into behavioral variant frontotemporal dementia (bvFTD) [6] and language variant primary progressive aphasia (PPA) which in its turn comprises three variants [7]. The most common genetic etiologies resulting in FTLD include mutations in tau (*MAPT*) and progranulin genes (*GRN*) and a repeat expansion in *C9orf72* [8-13]. Molecular pathologies underlying FTLD include aggregation from tau (FTLD-tau) or fused-in-sarcoma proteins (FTLD-FUS), accounting for approximately 45% and <5% of patients respectively [14]. The major pathological subtype, accounting for approximately 50% of FTLD population, is FTLD-TDP where patients have brain inclusions of transactive response DNA-binding protein of 43 kDa (TDP-43) [15,16]. Under physiological conditions, TDP-43 is a predominantly nuclear protein and its role in transcription and splicing regulation is well characterized [17]. In FTLD-TDP, TDP-43 is redistributed to the cytoplasm where it forms intraneuronal inclusions. This leads to an apparent loss of nuclear TDP-43 function, while the accumulation itself is expected to be toxic [18,19]. Furthermore, aggregation of TDP-43 is also characteristic for amyotrophic lateral sclerosis (ALS) [16], and clinical and genetic overlap between both disorders has corroborated their association in an FTLD-ALS spectrum [20]. Noteworthy, other neurodegenerative disorders can present with TDP-43 pathology as secondary feature, and this is the case in 20-50% of patients with AD and related tauopathies [14,21]. A comorbid TDP-43 pathology is reported to worsen neurodegeneration independently of AD pathology, leading to a more severe clinical presentation of dementia [22].

**Figure 1:**
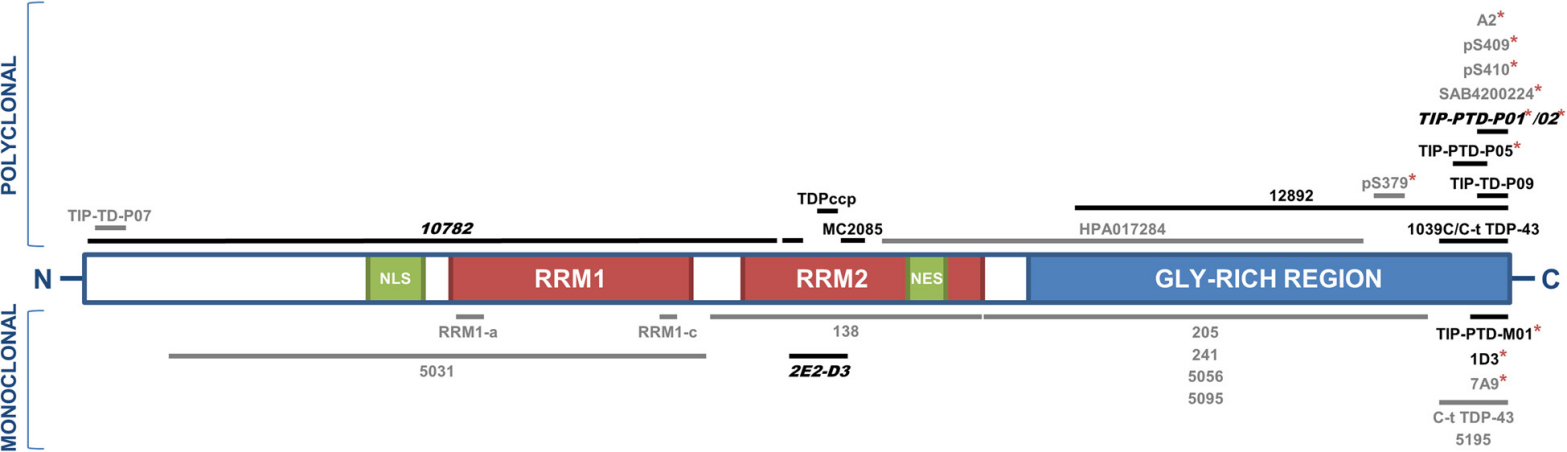
**Selected antibodies mapped to the TDP-43 protein.** Antibodies from the primary selection are marked in black, antibodies from the secondary selection are marked in grey. Asterisks indicate phosphorylation-dependent antibodies. Bold italic antibodies are those which are considered “standard” when studying TDP-43. NLS = nuclear localization signal, NES = nuclear export signal, RRM = RNA-recognition motive.

While mutations in a specific gene induce an associated molecular pathology, no strict relationship exists between clinical FTLD subtype and underlying proteinopathy [15,23]. Indeed, clinical symptoms rather reflect affected brain regions, which is especially exemplified in the heterogeneity of clinical FTLD. Moreover, it should be noted that up to 25% of clinical FTLD is actually due to atypical presentation of AD pathology [14,24]. The two-way clinicopathological association between FTLD(−TDP) and AD shows there is an urgent need for biomarkers that allow early and differential diagnosis of FTLD. A promising approach is quantification of disease-specific biochemical markers present in biofluids (cerebrospinal fluid (CSF) and blood) [23]. At present, well-characterized and validated diagnostic markers specific to FTLD pathology do not exist, with the exception of decreased progranulin concentrations for *GRN* mutation-related FTLD, a subgroup of FTLD-TDP [25,26]. A high-ranking candidate to become a biomarker for all FTLD-TDP patients is TDP-43 itself. Because of low absolute levels, quantitative analysis of TDP-43 in biofluids will demand a very sensitive immunoassay, preferably specific for pathological TDP-43 [27]. The TDP-43 protein comprises two RNA-recognition motives (RRM1-&2) and a glycine-rich C-terminal domain (Figure 1) [17,28]. Pathological aggregation of TDP-43 is regulated by both N-terminal and C-terminal regions, but also includes modifications like truncation, ubiquitination and phosphorylation [16,29-32]. Reported truncation sites are located inside the RRM2 and include Arg208, Asp218 and Asp247 [33-35] while major phosphorylation sites are serine residues located near the C-terminal end of TDP-43 [32,36].

The primary objective of this systematic review is to identify which antibodies have previously been described to detect TDP-43 pathology. These antibodies are expected to be suitable for defining the characteristic profile of pathological TDP-43 in human brain and biofluids, using immunostaining and immunoblotting. Additionally, antibodies specific for TDP-43 pathology hold potential to be used in immunoassays for quantification of TDP-43 and its pathological isoforms in biofluids. In this regard, a secondary objective is to review existing immunoassays with reference to specific antibody pairs.

## Methods

A systematic review of existing literature was performed by searching PubMed and Web of Science using the predefined keywords “TDP 43” and variations like “transactive response DNA binding protein” or “TAR DNA binding protein” in publication title, and keywords indicating the use of antibodies (such as “antibody”, “immunoblot”, “immunohistochemistry” “immunoassay” or “ELISA” or, to broaden the search, “aggregate” or “inclusion”) in title or abstract for PubMed and as topic for Web of Science. Only original research papers published between 06/10/2006 (first report of TDP-43 as pathological protein in disease [16]) and 31/12/2014 were considered. For each English research paper the methods section was checked to select papers able to answer our learning objective.

### Quality assessment

Papers that did not report the use of an antibody directed at TDP-43 (e.g. genetics based) were excluded. Because the aim was to assess specificity of antibodies with applicability to human disease, papers using yeast, *C. elegans* or *Drosophila* as model organism were excluded. From relevant papers following data was extracted and tabulated: antibody characteristics (i.e. name, epitope, manufacturer, clonality, species), used immunoassays, assessed target (e.g. organism, cell line, biofluid, tissue), relevant immunoassay outcomes (i.e. description of staining, biochemical profile, TDP-43 concentration) and study strengths and limitations. For completeness of the systematic review, antibodies were characterized thoroughly by consulting available published data, manufacturer websites and corresponding authors. This allowed identification of epitopes at amino acid (AA) level [37,38] and identification of monoclonal clone numbers.

When provided with enough information to identify the exact antibody (known name, epitope, manufacturer, clonality and species), published results were scored for each paper that used the same antibody both in immunostaining and immunoblotting experiments. The scoring is a basic “Yes/No” system indicating if an antibody reacts with pathological TDP-43 in the reported setup or not. For staining, a positive score signifies visualization of any form of pathological inclusions, while for blotting a positive score responds to the presence of high molecular weight smears and/or distinct C-terminal fragments [39,40]. A negative score was given when there was no reactivity or only nuclear TDP-43 of 43 kDa was described as immunoassay result. Scoring was performed independently by two researchers (JG, EVM) and discrepancies (<10%) were rescored by consensus.

From tabulated scores, a primary selection of antibodies was listed based on having a positive score on one or both immunoassays multiple times. A secondary selection was made from antibodies that scored positive on one or both immunoassays only once.

## Results

### Search results

The initial search resulted in a total of 671 publications, including 490 found in both databases. After selection of relevant English research papers 409 publications remained. These publications report the use of 833 antibodies (approximately 100 unique) from which the characteristics were extracted. Relevant immunoassay outcomes were scored for each well-characterized antibody (506 of 833 reports, 74 unique antibodies). In 132 of these reports both immunostaining and immunoblotting were performed with the same antibody, leaving a total of 38 different antibodies for further assessment.

### Main results

The primary selection, representing antibodies capable of detecting TDP-43 pathology with both immunoassays, includes 9 unique antibodies reported 62 times with a double positive score and 33 times with a single positive score. One additional antibody only has multiple reports with a single positive score (Table 1). The secondary selection consists of 19 unique antibodies (Additional file 1: Table S1).

**Table 1:** **List of antibodies that are selected based on their ability to detect TDP-43 pathology, reported multiple times**

**Antibody (Epitope/Immunogen)**	**Manufacturer/First report**	**Clonality**	**Species**	**Times reported with scores** ***1-1***	**Times reported with scores** ***0*** **–** ***1*** **or** ***1-0***	**Times reported with scores** ***0-0***	**Total times scored (n = 104)**
1D3 (pS409/410, AA 403–414/P)	Millipore/Neumann 2009	mono	rat	3	-	-	3
TIP-PTD-P05 (pS403/404; AA 398–408/P)	Cosmo Bio/Hasegawa 2008	poly	rabbit	5	1	-	6
TIP-PTD-M01 (pS409/410; AA 405–414/P)	Cosmo Bio/Inukai 2008	mono	mouse	7	2	-	9
***TIP-PTD-P01&-P02*** (pS409/410; AA 405–414/P)	Cosmo Bio/Hasegawa 2008	poly	rabbit	12	5	-	17
1039C/C-t TDP-43 (AA 394–414/P)	Igaz 2008	poly	rabbit	5	2	2	9
***10782*** (AA 203–209 and AA near N-terminus/R)	ProteinTech	poly	rabbit	14	9	2	25
***2E2-D3*** (AA 205–222/R)	Abnova, Novus, Abcam, etc.	mono	mouse	12	8	4	24
12892 (AA 288–414/R)	ProteinTech	poly	rabbit	3	2	1	6
TIP-TD-P09 (AA 405–414/P)	Cosmo Bio/Hasegawa 2008	poly	rabbit	1	2	-	3
MC2085 (AA 220–227/P)	Zhang 2009	poly	rabbit	-	2	-	2

Antibodies are ordered by percentage of double positive reports. Immunogen is depicted as P = peptide immunogen or R = recombinant protein immunogen. Bold italic antibodies are those which are considered “standard” when studying TDP-43.

A detailed look at the 10 highest-ranking antibodies (Table 1, Figure 1) shows that one antibody has two known epitopes, recognizing both the N-terminal region and the RRM2. Two other antibodies are directed at the RRM2, and three have an epitope located in the C-terminal region of TDP-43. The remaining four antibodies also map in the C-terminal region but are specific for the phosphorylated serine residues. A majority of antibodies are polyclonal (n = 7; monoclonal: n = 3), and most antibodies have been reported first as in-house generated (n = 7).

Regarding the supplementary 19 antibodies that have shown potential to detect TDP-43 (Additional file 1: Table S1, Figure 1), four have an epitope located in the N-terminal region. The other antibodies again map in the RRM2 (n = 2) or the C-terminal region (n = 12, of which six phosphorylation-specific). One antibody cannot be categorized accordingly, as its immunogen overlaps the RRM2 and C-terminal region. Other characteristics of the secondary selection are a balanced distribution of clonality (monoclonal: n = 9; polyclonal: n = 10) and a striking 16 antibodies originally in-house generated.

An interesting consideration in the context of this review is the reported use of enzyme-linked immunosorbent assays (ELISAs) to quantify TDP-43. A total of 10 publications are available, describing 12 assays [41-50]. However, only five different antibody pairs (i.e. five different assays) have been used, and two of these are commercially available kits (Table 2). Noteworthy, merely a single assay has been reported more than once. Evaluation of antibody pairs shows that three assays are completely made up of antibodies from the selection, which implies they are very specific for their respective targets. Regarding epitope specificity (and therefore ELISA target), two reported ELISAs, including the most commonly described one, use an antibody directed at the RRM2 of TDP-43 together with the antibody having epitopes in the N-terminal region and the RRM2. Consequently, these assays will quantify full-length TDP-43 and maybe, depending on the truncation site, some C-terminal fragments. Two other ELISAs quantify phosphorylated full-length TDP-43, by combining an antibody directed at the RRM2 (or at an unknown epitope) with an antibody recognizing the C-terminally located phosphorylated serine residues. Again, there is a possibility for the detection of some C-terminal fragments that have undergone phosphorylation depending on the truncation site within the RRM2. The last reported ELISA is specifically directed at the C-terminal region, and will therefore quantify all C-terminal fragments. Looking at the antibody specificity, it is reasonable to assume this assay also quantifies full-length TDP-43, while phosphorylated C-terminal fragments are more than likely excluded. However, this assay has only been reported with data on cell lysates and brain homogenates and has not been used in biofluids, let alone in a patient study [47].

**Table 2:** **Overview of existing ELISAs, with detailed information about used antibodies**

	**Antibody (Epitope)**	**Manufacturer/First report**	**Clonality**	**Species**	**Target detected**	**Times reported**	**References**
Capture	***2E2-D3*** (AA 205–222)	Abnova, Novus, Abcam, etc.	mono	mouse	• full length TDP-43	8	[41-45,48-50]
Detection	***10782*** (AA 203–209 and AA near N-terminus)	ProteinTech	poly	rabbit	• (some CTFs)		
Capture	***2E2-D3*** (AA 205–222)	Abnova, Novus, Abcam, etc.	mono	mouse	• pTDP-43	1	[42]
Detection	***TIP-PTD-P02*** (pS409/410, AA 405–414)	Cosmo Bio/Hasegawa 2008	poly	rabbit	• (some pCTFs)		
Capture	***10782*** (AA 203–209 and AA near N-terminus)	ProteinTech	poly	rabbit	• full-length TDP-43	1	[46]
Detection	60019 (AA 203–209)	ProteinTech	mono	mouse			
Capture	*not specified* ^*a*^	EIAab	poly	rabbit	• pTDP-43	1	[46]
Detection	SAB4200223 (pS409)^a^	Sigma	poly	rabbit	• (pCTFs)		
Capture	205 (AA 262–391)	Kwong 2014	mono	mouse	• (full length TDP-43)	1	[47]
Detection	1039C/C-t TDP-43 (AA 394–414)	Igaz 2008	poly	rabbit	• CTFs		
					• (pCTFs)		

^a^Information provided by kit manufacturer, confirming published assay details. Abbreviations: CTFs, C-terminal fragments; pTDP-43, phosphorylated TDP-43; pCTFs, phosphorylated C-terminal fragments. Bold italic antibodies are those which are considered “standard” when studying TDP-43.

Apart from aforementioned publications using ELISAs, only three other reports were found where TDP-43 is detected in biofluids, by means of an immunoblot [51-53].

## Discussion

This review was undertaken to identify the most suitable antibodies for future research of TDP-43, while evaluating ELISAs already used for its quantification. As much as 29 antibodies met the selection criteria and are thus reported to detect TDP-43 pathology. In contrast, only five different ELISAs have been published and their antibody combinations leave room for improvement.

The majority of antibodies in the final selection are characterized by an epitope in the C-terminal region, and many of them are specific for phosphorylated TDP-43. These findings are expected, since C-terminal truncation and phosphorylation are hallmarks of aggregated TDP-43 in disease [16,30,32]. However, the observed result is not bidirectional, as several antibodies directed at pathological isoforms do not specifically reflect TDP-43 pathology. This is the case for those antibodies that only obtained double negative scores, but is probably also the case for some antibodies in the secondary selection. The fact that certain antibodies have only been used once has two possible reasons. First, for “older” antibodies in the list, it might be an indication for their lack of sensitivity and specificity (false positives). This is a feature addressed by groups generating in-house antibodies themselves, who make a selection of antibodies to use for further research after internal validation [32,36]. With this in mind, it is not unlikely that antibodies of lower affinity are also generated and made available by commercial companies. Alternatively, “recent” antibodies in the secondary selection have not yet had the chance to be used multiple times (false negatives). However, also in these cases there can be antibodies that will not be pursued further by the manufacturing group [47,54]. Of note, the occurrence of false negatives because of a lack of replication studies also applies to antibodies that have not been used in both immunoassays in one publication and therefore were not included in the scored antibody list.

Another reason for false positive and false negative results is the fact that antibodies are always used in a very specific setup. For example, antibodies used for studying exogenous expressed C-terminal fragments are not necessarily successful in detecting pathological TDP-43 in patients. Conversely, when antibodies are used to study patients without suspected TDP-43 pathology it is understandable that they will indeed not detect pathology. These remarks are supported by the fact that many antibodies in the primary selection do not consistently have a double positive score, and some are even reported with a double negative score. Finally, there is the potential occurrence of biased results, since three antibodies are considered “standard” antibodies for studying TDP-43 proteinopathies, and are thus widely used. These antibodies are 10782 (ProteinTech), 2E2-D3 (available from different manufacturers) and TIP-PTD-P01&-P02 (Cosmo Bio) [38,55]. This assumption is reflected in the systematic review, as together they account for almost half the reports using a well-characterized antibody. 10782 (115/506 reports, 22.7%) and 2E2-D3 (68/506 reports, 13.4%) have been available since the beginning of TDP-43 studies [16,56]. TIP-PTD-P01&-P02 (68/506 reports, 13.4%) was first reported by the group which generated it, in a publication initiating the branch of research on phosphorylated TDP-43 [32]. Despite being commonly used, these antibodies are not necessarily the best options when studying TDP-43 pathology. 10782 only has a double positive score in 56.0% of its scored reports (14/25). However, it has been successful in the development of an ELISA both as capture or detection antibody, and is capable of immunoprecipitation. These facts prove that the antibody has the desired affinity for total TDP-43. 2E2-D3 is reported with a double positive score in 50.0% of its scored reports (12/24), and this antibody has also been successfully used in ELISAs and immunoprecipitation. Moreover, its specificity for human TDP-43 makes it very suitable in research of TDP-43 in transgenic animal models [38]. Nevertheless, when looking to study pathological TDP-43, TIP-PTD-P01&-P02 has a better track record, with 70.6% of its scored reports having a double positive score (12/17).

A final point of discussion when selecting antibodies to study TDP-43 is the use of commercially available antibodies or the generation of antibodies in-house. Despite favorable antigenicity profiling of TDP-43, only a few well-characterized antibodies within the systematic search are available from companies (n = 24, Additional file 1: Table S2). Most of these antibodies are polyclonal (n = 19) of which 15 are directed at the C-terminal region (including five phosphorylation-specific). Of the well-characterized commercial monoclonal antibodies one maps in the N-terminal region, two recognize epitopes in the RRM2 of TDP-43 and two are phosphorylation-dependent. The lack of available antibodies specific for pathology has stimulated the generation of novel antibodies. A number of groups have been successful using this approach and five in-house generated antibodies are now also commercially available [32,36,57]. However, many more have only been used a few times by the original group, in collaborative efforts or have not been used again after their first report [30,47,54,58-64]. Noteworthy, most high-ranking antibodies in the selection with an epitope directed at pathologically modified TDP-43 are originally in-house generated. From a research perspective this is a logical starting point when undertaking the production of antibodies, as these antibodies are more likely to be suitable for the study of TDP-43 pathology. As a side note, in-house generation of antibodies requires the use of either peptide immunogens or recombinant protein immunogens. In practice, only peptides can be used to obtain antibodies specific for major protein modifications. Indeed, all reported antibodies directed at phosphorylated TDP-43 within this search are generated using a peptide immunogen. The predominance of antibodies generated with peptide antigens is also related to the propensity of recombinant TDP-43 protein to aggregate, which is especially the case for C-terminal fragments [33]. With regard to ELISA development, antibodies generated with recombinant protein antigens are more than suitable when developing an ELISA for whole-target quantification, but when looking to quantify different isoforms of one protein optimal results are to be expected with peptide antigens.

As far as reported immunoassays detecting TDP-43 and its isoforms in biofluids go, it is clear that improvements can and should be made. Merely looking at used combinations of antibodies, previous ELISAs were developed to reflect either total TDP-43 or phosphorylated TDP-43 levels. However, none of these assays accounted for the presence of C-terminal fragments, as these may have been quantified together with full-length (phosphorylated) TDP-43. To utilize TDP-43 as an effective biomarker applicable for human TDP-43 proteinopathies, it is very likely that all isoforms of TDP-43 will need to be quantified separately. From this point of view, the ELISA reported by Kwong et al. [47] is already a step in the right direction, although it will probably still detect full-length TDP-43 together with C-terminal fragments. To solely quantify full-length TDP-43 a less ambiguous combination of antibodies should be used, that is certain to exclude any C-terminal fragments. The same requirement applies for quantification of phosphorylated full-length TDP-43 without interference from phosphorylated C-terminal fragments. Although lone detection of C-terminal fragments seems difficult, a few antibodies are reported to be specific enough to potentially achieve this [47,60], and further efforts should be undertaken to quantify this pathological isoform. When this is made possible, an assay specific for phosphorylated C-terminal fragments can also be developed, completing the spectrum of TDP-43 pathology.

In the context of this systematic review it is necessary to comment on the recent publication by Feneberg et al. describing a limited role for TDP-43 as a diagnostic tool [52]. The main finding of this publication is that TDP-43 present in CSF is mainly blood-derived. This is based on an immunoblot analysis, which shows that the concentration of TDP-43 in blood is 200 times higher than that in CSF. Based on published ELISAs it is difficult to comment on the comparison of TDP-43 levels between CSF and plasma. Only one study has quantified TDP-43 in CSF and plasma of the same patients and here only relative concentrations are reported, so no conclusion can be made with regard to blood/CSF ratio [46]. However, the claim that TDP-43 is mainly blood-derived and is therefore of minor importance as diagnostic tool is weak. Other studies on biomarker levels measured in paired blood and CSF samples demonstrate that correlations are more complex than anticipated. For example, though progranulin is much more abundant in plasma and its plasma and CSF levels correlate, plasma progranulin levels can only account for a very small part of the variability of CSF progranulin levels [65].

A final remark, which is also supported by Feneberg et al. [52], is that exosomes present in CSF may contain TDP-43 which is not blood-derived and may therefore be more suitable to reflect TDP-43 pathology. Thus, even if it is confirmed that TDP-43 in CSF has limited diagnostic value, this would only result in the adjustment of future immunoassays to another target, namely exosome preparations. But first, further evidence needs to be gathered by independent researchers before this assumption can be considered valid.

### Potential limitations of the study

In order to make sure selected antibodies can be used in all future studies of TDP-43 proteinopathies, regardless of preferred immunoassay, we decided to score only those antibodies used for both immunostaining and immunoblotting in the same publication. As this might be a methodological limitation of the review process, we performed the same analysis on the entire dataset which resulted in a comparable selection, including all 10 highest-ranking antibodies. Another possible limitation is the use of a scoring system to generalize descriptive results reported with different antibodies, under different experimental conditions. However, using a scoring system is indispensable in order to compare antibodies across studies, and all immunoassay outcomes were independently scored by two researchers to ensure reliable categorization.

## Conclusions

This systematic review provides an overview of antibodies reported to detect TDP-43 pathology. These antibodies can be used in animal models and patient studies of TDP-43 proteinopathies, such as FTLD. Thus, researchers studying these disorders can choose highly sensitive and specific antibodies most suitable for their objective based on this review. Antibodies from the primary selection have consistently proven their functionality in detecting pathological TDP-43 in both immunostaining and immunoblotting. Antibodies from the secondary selection have the potential to detect TDP-43, but validation of their results is necessary before conclusions can be made regarding their future use. Additionally, high-ranking antibodies are leading candidates when selecting an antibody pair in future development of novel quantitative immunoassays for TDP-43 in biofluids or exosome preparations, as a possible biomarker for FTLD-TDP.

## Additional file

Additional file 1: Table S1. List of antibodies that are selected based on their ability to detect TDP-43 pathology, reported once. **Table S2.** Alphabetical list of well-characterized commercially available antibodies within this systematic search.
