# Supplementary material for: TDP-43 as a possible biomarker for frontotemporal lobar degeneration: a systematic review of existing antibodies
Source: Acta Neuropathol Commun. 2015 Apr 1;3:15. doi: 10.1186/s40478-015-0195-1 (PMC4380254; doi:10.1186/s40478-015-0195-1)
Supplement: Additional file 1: Table S1. — List of antibodies that are selected based on their ability to detect TDP-43 pathology, reported once. Table S2. Alphabetical list of well-characterized commercially available antibodies within this systematic search. [file 40478_2015_195_MOESM1_ESM.pdf]

Additional file 1.pdf

Table S1 List of antibodies that are selected based on their ability to detect TDP-43 pathology, reported once

Table S2 Alphabetical list of well-characterized commercially available antibodies within this systematic search.

Table S1 List of antibodies that are selected based on their ability to detect TDP-43 pathology, reported once

**Antibody (Epitope / Immunogen)**	**Manufacturer/First report**	**Clonality**	**Species**	**Times reported with scores *1-1***	**Times reported with scores *0-1* or *1-0***	**Times reported with scores *0-0***	**Total times scored (n = 19)**
5056 (AA 262-391 / R)	Kwong 2014	mono	mouse	1		-	1
138 (AA 182-261 / R)	Kwong 2014	mono	mouse	-	1	-	1
205 (AA 262-391 / R)	Kwong 2014	mono	mouse	-	1	-	1
241 (AA 262-391 / R)	Kwong 2014	mono	mouse	-	1	-	1
5031 (AA 25-181 / R)	Kwong 2014	mono	mouse	-	1	-	1
5095 (AA 262-391/ R)	Kwong 2014	mono	mouse	-	1	-	1
5195 (AA 394-414 / R)	Kwong 2014	mono	mouse	-	1	-	1
RRM1-a (AA 108-116 / P)	Shodai 2013	poly	rabbit	1	-	-	1
RRM1-c (AA 167-172 / P)	Shodai 2013	poly	rabbit	1	-	-	1
SAB4200224 (pS410 / P)	Sigma	poly	rabbit	1	-	-	1
HPA017284 (AA 232-372 / R)	Sigma	poly	rabbit	1	-	-	1
TIP-TD-P07 (AA 3-12 / P)	Cosmo Bio	poly	rabbit	1	-	-	1
7A9 (ps409/410; AA 403-414 / P)	Neumann 2009	mono	rat	1	-	-	1
A2 (pS409/410; AA 404-414 / P)	Kadokura 2009	poly	rabbit	1	-	-	1
C-tTDP-43 (AA 394-414 / P)	Igaz 2009	mono	mouse	1	-	-	1
pS379 (AA 375-384 / P)	Hasegawa 2008	poly	rabbit	1	-	-	1
pS409 (AA 405-414 / P)	Hasegawa 2008	poly	rabbit	1	-	-	1
pS410 (AA 405-414 / P)	Hasegawa 2008	poly	rabbit	1	-	-	1
TDPccp (AA 213-219 / P)	Rohn 2008	poly	rabbit	1	-	-	1

Antibodies are ordered by publication year, number of double positive reports and alphabetically.

Table S2 Alphabetical list of well-characterized commercially available antibodies within this systematic search. This requires a known name, epitope, manufacturer, clonality and species

**Antibody (Epitope)**	**Manufacturer/First report**	**Clonality**	**Species**
10782 (AA 203-209 and AA near N-terminus)	ProteinTech	poly	rabbit
12892 (AA 288-414)	ProteinTech	poly	rabbit
1D3 (pS409/410, AA 403-414)	Millipore/Neumann 2009	mono	mouse
22309 (pS409/410)	ProteinTech	poly	rabbit
2E2-D3 (AA 205-222)	Abnova/Novus/Abcam/etc.	mono	mouse
3448S (AA surrounding G400)	Cell Signaling Technology	poly	rabbit
60019 (AA 203-209)	ProteinTech	mono	mouse
3H8 (AA 1-150)	Abcam/Encor/Millipore	mono	mouse
ab41881 (AA 350-414)	Abcam	poly	rabbit
ARP38942_T100 (AA 337-386)	Aviva	poly	rabbit
H00023435-A01 (AA 1-260)	Abnova	poly	mouse
HPA017284 (AA 232-372)	Sigma	poly	rabbit
NB110-55376 (AA 350-414)	Novus	poly	rabbit
PA5-17011 (AA surrounding G400)	ThermoScientific	poly	rabbit
PAB12006 (AA 350-414)	Abnova	poly	rabbit
SAB4200223 (pS409)	Sigma	poly	rabbit
SAB4200224 (pS410)	Sigma	poly	rabbit
T1580 (AA 355-369)	Sigma	poly	rabbit
T1705 (AA 181-198)	Sigma	poly	rabbit
TIP-PTD-M01 (pS409/410; AA 405-414)	Cosmo Bio/Inukai 2008	mono	mouse
TIP-PTD-P01 & -P02 (pS409/410; AA 405-414)	Cosmo Bio/Hasegawa 2008	poly	rabbit
TIP-PTD-P05 (pS403/404; AA 398-408)	Cosmo Bio/Hasegawa 2008	poly	rabbit
TIP-TD-P07 (AA 3-12)	Cosmo Bio	poly	rabbit
TIP-TD-P09 (AA 405-414)	Cosmo Bio/Hasegawa 2008	poly	rabbit

Bold italic antibodies are those which are considered “standard” when studying TDP-43
